# Indigenous excellence: confronting racism and complicity in peer review

**DOI:** 10.1093/heapro/daag117

**Published:** 2026-08-12

**Authors:** Raglan Maddox, Sarah Funnell, Michelle Kennedy, Janet Smylie

**Affiliations:** Bagumani (Modewa) Clan, Papua New Guinea; Yardhura Walani, National Centre for Epidemiology and Population Health, Australian National University College of Law, Governance and Policy, 54 Mills Road, Canberra, ACT 2602, Australia; Unceded Algonquin Territory, Ottawa, Ontario, Canada; Department of Family Medicine, Queen’s University, PO Bag 8888, Kingston, Ontario, Canada K7L 5E9; Wiradjuri; School of Medicine and Public Health, University of Newcastle, University Drive, Callaghan, NSW 2308, Australia; Hunter Medical Research Institute, Locked Bag 1000, Newcastle, NSW 2305, Australia; Red River Métis; Well Living House, Li Ka Shing Knowledge Institute, and Department of Family and Community Medicine, St. Michael’s Hospital–Unity Health Toronto, 30 Bond Street, Toronto, Ontario, Canada M5B 1W8; Dalla Lana School of Public Health and Department of Family and Community Medicine, Faculty of Medicine, University of Toronto, Toronto, Ontario, Canada

**Keywords:** Indigenous health, peer review, racism, academic publishing, epistemic justice, health promotion

## Abstract

Racism in peer review is a common, structurally embedded, and systemic problem. However, it is often framed as isolated acts of individual-level bias. Racism in peer review manifests across interconnected societal, system, and process levels, shaping who participates in knowledge production, what is deemed credible evidence, and whose work is published. This Indigenous-led Perspective examines racism in peer review through a three-layer framework: (1) societal, (2) system, and (3) process (decision-making and interpersonal), situating peer review as one domain within the broader problem of anti-Indigenous publication bias. Grounded in the United Nations Declaration on the Rights of Indigenous Peoples (UNDRIP), Indigenous Data Sovereignty, and lived experience, we integrate conceptual analysis with illustrative examples to identify intervention points. At the societal level, colonial structures and socio-political forces determine whose knowledges count. At the system level, editorial governance, publishing models, and reward structures reproduce inequities. At the process (decision-making and interpersonal) level, racism cuts across reviewer selection and feedback, editorial decision-making, and dissemination through racialized assumptions, assessment and evaluative practices. We propose concrete, systemic reforms to dismantle racism in peer review, including structural policy reform, diversification of reviewer and editorial pools, anti-racism and cultural safety training, and explicit guidelines. We also outline (co)author responsibilities to act in solidarity when racism occurs. Equity, justice, and fairness in peer review demand more than goodwill. They require enforceable structural change, accountability, and culturally safe practice. Confronting racism is both an ethical imperative and a prerequisite for research excellence, integrity, and health promotion that serves all communities.

Contribution to Health Promotion

- Racism in peer review undermines scholarship, distorts the evidence base, and perpetuates inequities in research and policy.
- This Perspective introduces a three-layer framework to identify structural drivers of racism and bias within peer review: (1) societal, (2) system, and (3) process.
- Demonstrates how racialized evidence hierarchies shape what knowledges are legitimized, influencing research agendas, policy decisions, and health promotion practice.
- Proposes reforms grounded in Indigenous sovereignty, anti-racism, and research integrity to strengthen cultural safety, accountability, and justice in publishing.
- Strengthening peer review can expand the diversity of knowledges informing health promotion and support more ethical, inclusive, and effective public health action.

## Introduction

This Perspective is based on the lived and living experiences of four Indigenous health researchers and academics from different countries and Nations, with representation from what is called Australia, Papua New Guinea, and Canada (see Foundational to this Perspective). This paper is an Indigenous-led conceptual and critical Perspective grounded in lived experience, existing literature, and applied practice insights. Our Perspective focuses specifically on the peer review of manuscripts submitted by Indigenous health researchers to journals as a critical site where anti-Indigenous racism is produced, reproduced, and can be interrupted. While we acknowledge important overlaps with the peer review of research grants, conference abstracts, ethics review processes (including human research ethics committees), and institutional funding decisions, these domains are not the primary focus of this paper. Instead, we concentrate on the peer review process, recognizing it as one domain within a wider system of institutionalized, epistemic, and colonial power that governs academic legitimacy and systematically produces harmful experiences of anti-Indigenous racism. Peer review functions as a critical upstream determinant of the evidence base that shapes health promotion policy, practice, and resource allocation.

In using the term ‘anti-Indigenous publication bias’, we do not refer to the conventional epidemiological definition of publication bias, which usually describes the selective non-publication of studies based on the direction, magnitude, or statistical significance of findings. Rather, we use this term to describe a structural pattern within academic publishing systems.

In this context, anti-Indigenous publication bias occurs when Indigenous-led, Indigenous-governed, Indigenous-theorized, or Indigenous-specific scholarship is systematically disadvantaged, devalued, delayed, (re)shaped, or excluded. This does not occur solely because of the research findings themselves, but through racism, colonial epistemic hierarchies, and institutional practices embedded within journal norms, peer review processes, and editorial decision-making. Taken together, these structures shape how Indigenous knowledges are interpreted, evaluated, and judged as legitimate within academic publishing and the academy and society more broadly.

We acknowledge, but do not seek to comprehensively address the broader structural forces shaping academic publishing, including the political economy of health sciences journals, the increasing corporatization and profit-orientation of publishing, and the ways in which capitalist logics shape editorial priorities and knowledge valuation (Legg *et al*. 2021). While these forces are deeply implicated in anti-Indigenous publication bias, they fall beyond the scope of this Perspective. Further, this Perspective does not seek to adjudicate individual cases or attribute intent but rather examines how structural features of academic publishing produce patterned harms, irrespective of individual motivation (Legg *et al*. 2021, Lee *et al*. 2023).

### Racism in peer review: existence and influence

Our experiences have shown us that contemporary research and academic publishing are dominated by Whiteness, with Euro-Western values shaping what is recognized as evidence, how evidence hierarchies are constructed, and whose knowledges are valued and mobilized (Lee *et al*. 2023). These conditions have produced, and continue to produce, unsafe and unethical research designs, practices, and publications that actively oppress Indigenous peoples, racialized peoples, and their respective ways of knowing, being, and doing (Lee *et al*. 2023).

These outcomes are not abnormalities, but predictable consequences of systems designed to centre and protect Whiteness and Euro-Western norms (Lee *et al*. 2023). Processes that are often positioned as objective and neutral instead function as mechanisms through which colonial knowledge hierarchies are maintained, structurally advantaging Euro-Western epistemologies and methodologies while marginalizing Indigenous and racialized knowledge systems (Lee *et al*. 2023).

**Figure 1:**
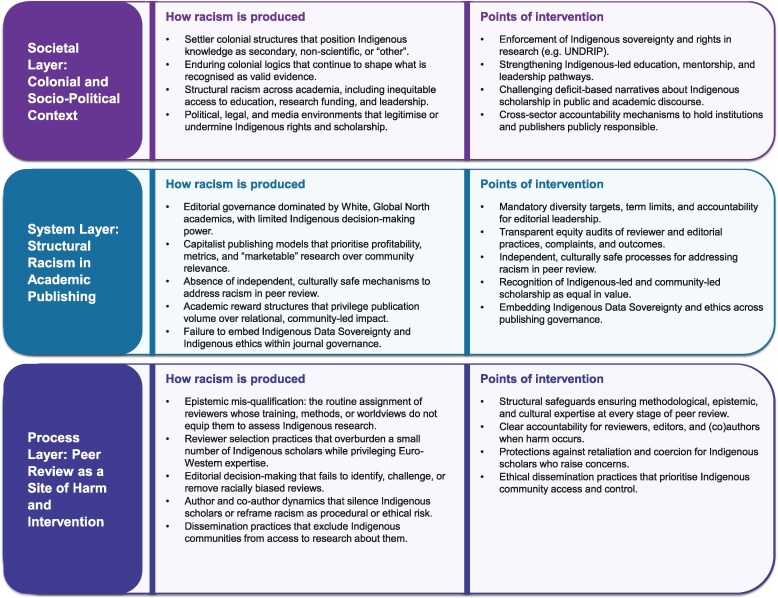
Racism in peer review: a three-layer framework—societal, system, and process, with points of intervention.

Against this backdrop, we examine racism in peer review across three interconnected layers: (1) societal, (2) system, and (3) process (decision-making and interpersonal), situating peer review as one specific domain within the broader pattern of anti-Indigenous publication bias (Fig. 1; Paradies 2006, Ahuriri-Driscoll *et al*. 2022, Piper *et al*. 2022, Lee *et al*. 2023). In this paper, we define ‘anti-Indigenous publication bias’ as the systematic privileging of Euro-Western epistemologies, methodologies, and authorship norms within academic publishing, which results in the disproportionate scrutiny, marginalization, or reframing of Indigenous research and researchers (Lee *et al*. 2023). Importantly, this process begins prior to external peer review, at the stage of initial editorial screening or ‘desk review’, where editors determine whether a manuscript is sufficiently aligned with the journal’s scope, standards, and perceived audience to be sent out for review (Legg *et al*. 2021, Lee *et al*. 2023). This early-stage filtering represents one of the least visible yet most powerful mechanisms of exclusion. Many submissions are rejected at this stage, often without transparent criteria or detailed feedback. While desk screening is framed as a quality and fit assessment, it is not immune to bias. Editorial judgements about novelty, rigour, relevance, or ‘generalizability’ may be shaped by dominant disciplinary norms and epistemic hierarchies that marginalize Indigenous methodologies, community-led research, and critical race analyses (Ware and Mabe 2015, Lee *et al*. 2023). We therefore examine specific processes across the publication pathway, from the selection of ‘appropriate’ peer reviewers to the biases reviewers bring to their assessments through to editorial interpretation and follow-up, author responses, and the dissemination of published work (Ware and Mabe 2015, Legg *et al*. 2021, Lee *et al*. 2023).

While this Perspective focuses specifically on anti-Indigenous racism, the three-layer framework may also have broader utility for understanding and addressing other forms of exclusion, marginalization, and bias within academic publishing, including those experienced by racialized, disabled, LGBTQIA+, and other excluded scholars.

While peer review is only one site through which anti-Indigenous publication bias operates, it represents a strategic point of intervention because it directly shapes whose knowledges are legitimized, amplified, or excluded from the scholarly record (Ware and Mabe 2015, Lee *et al*. 2023). Racism in peer review manifests in multiple forms, from everyday microaggressions and implicit bias to overt and explicit acts of discrimination. Regardless of form, intensity, or intent, all racism causes harm, undermines scholarship, and erodes trust in academic systems (Smith 2021, Watego 2021a, 2021b, Lee *et al*. 2023).

Racism in peer review is cumulative and systemic, operating across societal, system, and process levels. Effective reform requires coordinated intervention across all three layers (Katz *et al*. 2020, Watego 2021a, 2021b, Lee *et al*. 2023, Strauss *et al*. 2023).

## Societal layer—colonial and socio-political context

Upholding the academic and peer review system and process layers are the societal conditions shaped by settler colonialism and socio-political structures (Smith 2021, Watego 2021a, 2021b, Lee *et al*. 2023, Strauss *et al*. 2023). These are not abstract forces. They actively shape the terms of knowledge production, determining whose expertise is valued and whose is excluded. Settler colonial structures continue to position Indigenous knowledges as secondary or ‘othered’, sustained by historical, ongoing logics that continue to influence what is considered credible evidence (Smith 2021, Watego 2021a, 2021b, Lee *et al*. 2023). Broader academic inequities limit Indigenous access to education, research funding, and leadership, while political climates, laws, media narratives and popular discourse often undermine Indigenous sovereignty and self-determination (Smith 2021, Lee *et al*. 2023).

To address these societal drivers of anti-Indigenous racism in peer review, interventions must be structural and intersectional (Legg *et al*. 2021, Lee *et al*. 2023, Strauss *et al*. 2023, Maddox and Morton Ninomiya 2025). These interventions include enforcing Indigenous sovereignty and rights in research in line with United Nations Declaration on the Rights of Indigenous Peoples (UNDRIP) (United Nations 2007).

Strengthening Indigenous-led education and mentorship pathways; challenging deficit-based narratives about Indigenous scholarship; and building cross-sector alliances to hold publishers, institutions, funders, and people in positions of power accountable for upholding racist knowledge systems (Legg *et al*. 2021, Smith 2021, Lee *et al*. 2023, Strauss *et al*. 2023, Maddox and Morton Ninomiya 2025). These actions recognize that anti-Indigenous racism in academic publishing is inseparable from other intersecting systems of oppression and must therefore be addressed through approaches that are explicitly intersectional, accountable, and structurally focused (Lee *et al*. 2023, Maddox and Morton Ninomiya 2025).

These forces shape the policies, governance structures, and editorial cultures of academic publishing, which in turn determine how racism manifests at the system level (Lee *et al*. 2023, Maddox and Morton Ninomiya 2025).

## System layer—structural racism in academic publishing

Racist outcomes in peer review are not solely the result of individual bias; they are also produced by the structures of academic publishing. Editorial boards are often dominated by scholars from the Global North, with limited Indigenous and racialized representation, including in decision-making roles (Lee *et al*. 2013, 2023, Strauss *et al*. 2023, Maddox and Morton Ninomiya 2025, Sirleaf 2025). Further, Indigenous roles are often unpaid, junior, advisory, isolated, and rarely structurally funded. Profit-driven publishing models privilege ‘marketable’ research aligned with Euro-Western epistemologies, often sidelining Indigenous priorities. The absence of independent, culturally safe mechanisms to address racism in peer review, combined with limited transparency on reviewer demographics and bias outcomes, reinforces a lack of active accountability (Smith 2021, Walter and Suina 2023).

These inequities are compounded by reward structures that prioritize volume (‘publish or perish’) and impact factor over community-led, relational scholarship, and by the near-total absence of Indigenous Data Sovereignty and Indigenous ethics in publishing governance (Lovett *et al*. 2019, Walter and Suina 2023). System-level change demands structural interventions: mandating diversity targets and term limits for editorial boards; publishing annual equity audits; establishing independent review panels to address racism-related complaints; decoupling academic recognition from volume-based metrics; and embedding Indigenous governance principles into all stages of editorial decision-making (Lovett *et al*. 2019, Lee *et al*. 2023, Walter and Suina 2023).

Indigenous-led and community-controlled journals, such as First Nations Health and Wellbeing—The Lowitja Journal, demonstrate how Indigenous governance, epistemic leadership, and community accountability can be embedded within publishing structures, offering concrete alternatives to dominant Euro-Western models (Lovett *et al*. 2019, Lee *et al*. 2023, The Lowitja Journal 2026).

## Process layer—racism in peer review: steps, harms, and interventions

At the process level, racism enters peer review through a series of sequential and interrelated stages. Importantly, these processes begin before formal peer review, at the point of initial editorial screening or ‘desk review’, where editors determine whether a submitted manuscript proceeds to external review. For analytic clarity, we examine these stages as: (i) initial editorial assessment and triage; (ii) reviewer selection; (iii) the epistemic lenses, biases, and gaps in expertise that reviewers bring to the assessment of Indigenous research; (iv) editorial interpretation and oversight of peer review reports; (v) editorial communication with authors; (vi) author responses to peer review, including co-author dynamics; and (vii) publication, dissemination, and community access to findings (Helmer *et al*. 2017, Tennant *et al*. 2017, Smith 2021, Strauss *et al*. 2023, Maddox and Morton Ninomiya 2025). While these stages are presented sequentially, they are mutually reinforcing and shaped by the broader societal and system-level conditions described above (Lee *et al*. 2013, 2023, Maddox and Morton Ninomiya 2025).

Peer review is often presented as an objective, unbiased, merit-based process, including at the editorial screening stage, but racism can and does enter at every point of manuscript assessment, from initial editorial decisions about ‘fit’, ‘scope’, or ‘rigour’, through to reviewer selection and the dissemination of published work (Lee *et al*. 2013, Helmer *et al*. 2017, Smith 2021, Maddox and Morton Ninomiya 2025). This layer examines the practical, step-by-step processes where inequities emerge and can be interrupted. The type of racism, whether implicit or explicit, everyday, or overtly hostile, does not lessen its impact. All forms of racism are harmful, erode trust, and damage the integrity of the academic record (Paradies 2006, Watego 2021a, 2021b, Ahuriri-Driscoll *et al*. 2022, Strauss *et al*. 2023).

Having mapped the process-level operation of racism in peer review, we note that addressing racism within peer review and associated editorial decision-making processes is one necessary intervention in dismantling anti-Indigenous publication bias, but it is not sufficient on its own (Paradies 2006, Ahuriri-Driscoll *et al*. 2022, Strauss *et al*. 2023). While peer review is a site of intervention, it is only one domain within a broader ecology of anti-Indigenous publication bias that includes journal ownership structures, profit-driven publishing models, and the commodification of Indigenous suffering within health research. These broader dynamics warrant dedicated analysis beyond the scope of this Perspective (Paradies 2006, Ahuriri-Driscoll *et al*. 2022, Lee *et al*. 2023, Strauss *et al*. 2023).

An additional and often overlooked site of harm occurs after a manuscript has been accepted, during copy editing and production (Tennant *et al*. 2017, Smith 2021, Strauss *et al*. 2023). In our experience, Indigenous authors can experience the removal, alteration, or suppression of Indigenous identifiers, including Indigenous affiliation, community identification, and self-located positionality within author by-lines or affiliations (Tennant *et al*. 2017, Smith 2021, Strauss *et al*. 2023). These removals may occur despite repeated author requests and formal correspondence insisting on their inclusion.

Such interventions represent a form of epistemic and identity-based erasure, where Indigenous authorship is rendered abstracted from Country, Nationhood, and sovereignty in the final published record, and may be suppressed by Euro-Western academic institutions (Smith 2021, Strauss *et al*. 2023, Maddox and Morton Ninomiya 2025).

Importantly, these harms occur even in manuscripts that are Indigenous-led or authored predominantly by Indigenous scholars, demonstrating that Indigenous authorship alone does not protect against racialized editorial power once control shifts to publishers and production teams (Smith 2021, Strauss *et al*. 2023).

The routine treatment of Indigenous Nation identifiers as optional, stylistic, or negotiable reflects a deeper failure within publishing systems to recognize Indigenous identity and sovereignty as integral to scholarly legitimacy rather than ancillary metadata. When journals remove or resist Indigenous self-identification at the production stage, they (re)produce colonial logics that detach knowledges from people and place, undermine Indigenous Data Sovereignty, and compromise authorship integrity at the point of dissemination (Lee *et al*. 2023).

Preventing this form of harm requires journals to adopt explicit, enforceable policies that protect Indigenous author self-identification, including Nation affiliation, across all stages of publication, from submission through peer review to final copy-editing and production (Lee *et al*. 2023, Strauss *et al*. 2023). These identifiers should be treated as non-negotiable elements of authorship integrity, not discretionary editorial preferences (Lee *et al*. 2023, Strauss *et al*. 2023, Maddox and Morton Ninomiya 2025).

Table 1 operationalizes racism in peer review by mapping specific process stages to forms of harm and actionable interventions. It complements Fig. 1, which provides a conceptual, multi-level framework, by foregrounding where and how intervention is possible in practice.

**Table 1:** Racism in peer review: process stages, harms, and points of intervention.

Peer review stage	How racism manifests	Resulting harms	Intervention points (what changes practice)
**Reviewer selection**	Narrow, homogeneous reviewer pools Over-reliance on a small number of Indigenous reviewers (Colonial Load/minority tax) Tokenistic inclusion of a single Indigenous reviewer Assignment of reviewers without Indigenous methodological, ethical, or epistemic competence	Epistemic mis-qualification Normalization of inappropriate critique Overburdening Indigenous scholars Early delegitimization of Indigenous methodologies	Transparent reviewer selection policies prioritizing epistemic, methodological, and cultural expertise Expanded reviewer databases including Indigenous scholars and community experts (with remuneration) Routine tracking of reviewer workload and demographics Mandatory reviewer self-location and competence statements Routine consideration of author-nominated reviewers with appropriate expertise
**Review process/reviewer lens**	Indigenous methods framed as ‘weak’, ‘unscientific’, or ‘out of scope’ Reviewer unfamiliarity miscast as author deficiency Enforcement of Euro-Western epistemic norms	Deficit framing of Indigenous research Epistemic violence Reproduction of Whitewashed evidence hierarchies	Mandatory anti-racism, cultural safety, and Indigenous methodologies training Editorial recognition of epistemic mis-qualification (reviewer limitation, not author flaw) Pairing reviewers with complementary epistemic expertise
**Editorial review of peer review reports**	Failure to identify or challenge racist or deficit-based comments Deference to reviewer authority under claims of ‘objectivity’ or ‘academic freedom’	Harmful reviews legitimized Racism normalized through editorial silence Author safety undermined	Culturally safe editorial screening protocols Clear editorial authority to reject, revise, or replace biased reviews Indigenous and racialized editors in decision-making roles
**Editorial–author communication**	Editorial summaries that reproduce biased reviewer framing	Re-traumatization of authors	Respectful, transparent communication that names harm
	Minimization or proceduralization of harm	Silencing of racism concerns Erosion of trust in publishing systems	Protected pathways for authors to raise racism concerns without penalty
**Author response dynamics**	(Co)authors discouraging the naming of racism	Complicity in harm	Explicit author accountability guidelines
	Racism reframed as ‘ethical’ or ‘integrity’ risk	Reinforcement of Whiteness and power hierarchies	Journal recognition of coercion, retaliation, or silencing within author teams
	Institutional self-protection prioritized over Indigenous safety	Loss of Indigenous leadership and authorship	
**Publication, dissemination, and community access**	Indigenous communities not receiving published research	Breach of Indigenous Data Sovereignty	Mandatory return of research outputs to communities
	Secondary data use without consent or accountability	Extractive knowledge production	Enforcement of Indigenous data Sovereignty principles Open access for Indigenous-led research
		Ongoing colonial harm	

Racism in peer review is cumulative and process-driven. Effective reform, therefore, requires stage-specific interventions, rather than reliance on goodwill, individual complaint, or *post hoc* ethics processes (Paradies 2006, Piper *et al*. 2022, Lee *et al*. 2023, Strauss *et al*. 2023, Maddox and Morton Ninomiya 2025, The Lowitja Journal 2026).

## Peer review models and epistemic location

Peer review is presented as a mechanism for quality control, assisting researchers to strengthen their work, enhance rigour, and enable audiences to trust published findings (Legg *et al*. 2021, Lee *et al*. 2023, Strauss *et al*. 2023). However, beyond this technical role, it shapes which narratives are elevated within scientific discourse, including in Indigenous health and wellbeing. This process is neither neutral nor objective. It is shaped by historical, political, and epistemic power. Peer review operates in a racialized system that advantages Euro-Western knowledges and marginalizes Indigenous and racialized scholarship (Lee *et al*. 2023, Strauss *et al*. 2023).

The experiences, lenses, and biases that reviewers bring to the review process are critical to how manuscripts are assessed (Helmer *et al*. 2017, Katz *et al*. 2020, Lee *et al*. 2023, Strauss *et al*. 2023). Depending on academic discipline, reviewers have varying capacity and training to recognize, articulate, and critically reflect on the worldview or epistemological assumptions they bring to peer review, or to recognize and respect distinct epistemes (Lee *et al*. 2013, 2023, Helmer *et al*. 2017, Strauss *et al*. 2023). These limitations extend beyond reviewers to editorial decision-makers, many of whom may lack the cultural, epistemic, or racial literacy required to identify when reviewer comments (re)produce racism or colonial assumptions (Lee *et al*. 2013, 2023, Helmer *et al*. 2017, Strauss *et al*. 2023). Reviewers often assess research based on frameworks shaped by Euro-Western knowledge systems, including discipline-specific norms that are treated as universal or objective, which may consciously or subconsciously marginalize Indigenous knowledges and approaches (Lee *et al*. 2013, 2023, Helmer *et al*. 2017, Strauss *et al*. 2023). For example, in quantitative health sciences, positivist epistemologies and linked assumptions about evidence, validity, and rigour are commonly and implicitly assumed, rendering Indigenous methodologies, relational approaches, and alternative knowledge systems as ‘less rigorous’ (Smith 2021, Strauss *et al*. 2023).

Importantly, the criteria used to assess evidence, validity, rigour, and quality are themselves socially and historically constructed rather than universal or value-neutral. Indigenous scholars have argued that evidence-based practice (EBP) frameworks frequently privilege Euro-Western assumptions about what constitutes legitimate knowledge and expertise, while marginalizing Indigenous ways of knowing, doing, and being (Luke *et al*. 2022, Lee *et al*. 2023). As a result, peer review may reproduce epistemic exclusion not only through reviewer bias, but through the underlying standards used to judge research quality itself (Luke *et al*. 2022).

Dominant peer review models, particularly single- and double-blind review, are frequently positioned as safeguards of objectivity and fairness. However, these models also obscure opportunities for relationality, reciprocity, and social accountability that are foundational to many Indigenous research paradigms (Smith 2021, Lee *et al*. 2023, Strauss *et al*. 2023). By anonymizing relationships and removing context, blind review can limit the capacity for reviewers to be accountable to communities, to recognize relational obligations embedded in Indigenous research, or to engage ethically with lived experience and place-based knowledges. In practice, this often results in Indigenous authors being placed in the position of having to identify, explain, and justify why particular reviewer comments are racist, inappropriate, or epistemically misaligned, labour that is rarely recognized and disproportionately borne by Indigenous scholars (Smith 2021, Lee *et al*. 2023, Strauss *et al*. 2023).

Peer review is also structurally non-relational. It typically occurs without face-to-face interaction, dialogue, or embodied accountability between reviewers and authors. This distance can create a form of procedural coldness in which reviewers may not fully appreciate the relational, emotional, or professional consequences of their language (Moreton-Robinson 2017, Smith 2021, Strauss *et al*. 2023). Anonymity and institutional mediation can reduce opportunities for reflexive engagement, repair, or mutual learning. In contrast, relational encounters, including structured dialogue about harmful reviews, can create moments of growth, accountability, and changed practice (Lee *et al*. 2013, Moreton-Robinson 2017, Smith 2021, Strauss *et al*. 2023, Maddox and Morton Ninomiya 2025). Indigenous research paradigms emphasize relational responsibility, reciprocity, and accountability to people and community (Moreton-Robinson 2017, Smith 2021). When peer review systems strip away relational context, they risk enabling harm that would be less likely in accountable, dialogical settings. Structured, relational reflection on harmful reviews may have demonstrated transformative potential for reviewers and editors, suggesting that accountability increases when relational presence is restored (Moreton-Robinson 2017, Smith 2021, Strauss *et al*. 2023).

To help mitigate this, some journals are introducing bias training, cultural safety programmes, and reflexive practices that encourage reviewers to explicitly consider their positionality, epistemological standpoint, power, and privilege (The Lowitja Journal 2026). Structured guidelines or checklists that prompt reviewers to consider Indigenous knowledges and cultural relevance have been developed, helping to standardize an inclusive, respectful, and safe approach (The Lowitja Journal 2026). However, without parallel expectations and training for editors, responsibility for recognizing and responding to racism often remains implicitly shifted onto Indigenous authors rather than embedded within editorial accountability. Explicit reviewer self-location, including acknowledgement of positionality, power, and epistemic standpoint in relation to the research and communities under review, should be standard practice in peer review, particularly for Indigenous-focused scholarship. In some contexts, open or transparent peer review models have also been proposed as mechanisms to support greater accountability, dialogue, and ethical responsibility, though such approaches require careful implementation to avoid unintended harms (Moreton-Robinson 2017, The Lowitja Journal 2026).

While these processes are essential to improving the peer review of articles, they are only one aspect of a larger and often problematic system within academic health sciences publishing. Increasingly, journals operate within capitalist frameworks that prioritize metrics, profitability, and impact factors (Paradies 2006, Lee *et al*. 2013, Helmer *et al*. 2017, Legg *et al*. 2021, Strauss *et al*. 2023). This model can conflict with the ethical responsibility of being accountable to the communities represented within research. As a result, scientific careers are often advanced ‘on the backs of the ill’, especially in fields addressing Indigenous health and wellbeing, where Indigenous Knowledge Translation (KT) is often overshadowed by settler language, discourse and norms (Paradies 2006, Lee *et al*. 2013, Legg *et al*. 2021, Strauss *et al*. 2023). This broader issue of anti-Indigenous publication bias, of which peer review is only one domain, highlights the need for systemic reforms that prioritize inclusivity, accountability, and equity within academic publishing (Smith 2021, Lee *et al*. 2023, Strauss *et al*. 2023, Maddox and Morton Ninomiya 2025).

Peer review processes vary, being single-blind, double-blind, open, or transparent (Table 2). These can assess manuscripts and proposals, conference papers and other documents based on a range of criteria, such as academic rigour, accuracy, originality, validity, and significance (Legg *et al*. 2021, Smith 2021, Strauss *et al*. 2023). Recognizing the strengths and limitations of different peer review models is an important step towards more equitable and ethical scientific discourse. This includes attention to their alignment or misalignment with Indigenous values of relationality, reciprocity, and accountability, even as larger structural challenges within academic publishing remain (Moreton-Robinson 2017, Smith 2021, Lee *et al*. 2023).

**Table 2:** Types of peer review.

Peer review model	Description
**Single-blind**	Peer reviewers know the names of the authors, but the authors do not know who reviewed their manuscript unless the reviewer chooses to sign their report.
**Double-blind**	Peer reviewers do not know the names of the authors, and the authors do not know who reviewed their manuscript.
**Open peer review**	Authors know who the peer reviewers are, and the reviewers know who the authors are. If the manuscript is accepted, the named reviewer reports are published alongside the article and the authors’ response to the reviewer.
**Transparent peer review**	Peer reviewers know the names of the authors, but the authors do not know who reviewed their manuscript unless the reviewer chooses to sign their report. If the manuscript is accepted, the anonymous reviewer reports are published alongside the article and the authors’ response to the reviewer.

For example, open and non-blinded peer review models raise distinct ‘conflict-of-interest’ considerations, particularly in Indigenous research contexts where scholarly communities are small, relational, and highly interconnected (Moreton-Robinson 2017, Smith 2021, Lee *et al*. 2023). Euro-Western conflict-of-interest frameworks are typically premised on anonymity, distance, and individualized professional relationships and may not translate cleanly to Indigenous knowledge systems grounded in relationality, collective responsibility, and ongoing accountability (Lee *et al*. 2013, 2023, Moreton-Robinson 2017, Smith 2021, Strauss *et al*. 2023).

## Whitewashed evidence base

There is underrepresentation and misrepresentation of Indigenous knowledges in health sciences academia (Smith 2021, Lee *et al*. 2023). This includes Indigenous ways of knowing, being, and doing in academic literature, ultimately leading to a peer-reviewed evidence base that is Whitewashed through colonial knowledge infrastructure (Smith 2021, Lee *et al*. 2023). Whitewashing is a process of structural and systemic discrimination, racism, and exclusion that actively alters or omits Indigenous and non-Euro-Western contributions and perspectives to fit the dominant Euro-Western norms (Smith 2021, Lee *et al*. 2023). Racialized logics, produced by White Euro-Western institutions of socio-political and economic power, centre White Euro-Western knowledges and were historically manufactured to establish the racial ‘superiority’ of White people (Lee *et al*. 2023). Originating in the 1600s and solidified by eighteenth-century scholars, the concept of race emerged to justify the *trans*-Atlantic slave trade, treating humans as commodities. This racial capitalism, marked by physical and structural violence, persists today, perpetuating hierarchical notions used to legitimize colonization (Lee *et al*. 2023). These historical logics are not relics but underpin present-day academic gatekeeping, determining whose knowledges count and whose knowledges are excluded (Smith 2021, Lee *et al*. 2023, Sirleaf 2025).

Some of the theories, mechanisms, and science used to establish racial ‘superiority’ have been rejected and discredited (Lee *et al*. 2023). However, these legacies continue to influence scientific discourse today. For example, a quintessential form of racialized logic is to ‘default’ to a White reference group when considering race or ethnicity, regardless of the research question or scope. Applications of this approach include ‘race norming’, ‘race conditioning’, ‘race correction’, or ‘ethnic adjustment’ (Piper *et al*. 2022, Lee *et al*. 2023). These default practices embed racial biases into study designs and outcomes, often framing Indigenous and non-White populations as ‘deviant’ from White norms. This logic permeates the entire peer review process, influencing each step from reviewer selection to feedback, ultimately contributing to the underrepresentation of Indigenous knowledges and methodologies (Watego 2021a, 2021b, Piper *et al*. 2022, Lee *et al*. 2023).

## Peer review process—stepwise illumination of inherent racism (Table 1)

A critical stepwise examination of the peer review process across the stages of manuscript processing quickly reveals cross-cutting inherent racism (see Table 1) (Strauss *et al*. 2023).

### Reviewer selection

Peer reviewers are typically chosen based on their subject-matter expertise and familiarity with specific methodologies and methods, which often privilege Euro-Western, Eurocentric frameworks (Strauss *et al*. 2023). In practice, this frequently results in priority being given to Euro-Western research designs, such as randomized controlled trials (RCTs) and positivist intervention models, over Indigenous methodologies, relational approaches, and community-governed designs, including in areas such as smoking cessation research (Smith 2021, Lee *et al*. 2023, Strauss *et al*. 2023). This results in Indigenous methodologies and methods being reviewed by individuals who may lack understanding of non-Euro-Western approaches, leading to dismissive and/or racist feedback (Smith 2021, Lee *et al*. 2023, Strauss *et al*. 2023).

Bias in peer review selection also impedes progress in the pursuit of research and academic excellence, including the active selection and exclusion of specific peer reviewers. When the pool of reviewers is limited and homogeneous, it can normalize and perpetuate racism and racialized biases (Lee *et al*. 2013, Smith 2021, Strauss *et al*. 2023).

This reflects the historical and ongoing exclusion of Indigenous peoples from academic education, driven by colonial policies in Australia, Aotearoa New Zealand, Canada, and the United States, among other countries (Truth and Reconciliation Commission of Canada 2015, Hayward *et al*. 2021). Many instances of racial bias in review are less about personal temperament and more about a structural gap in reviewer expertise, training, and qualification. Nevertheless, journals continue to assign reviewers whose expertise aligns with Euro-Western paradigms to Indigenous-led and Indigenous-governed research, implicitly treating Euro-Western methodological training as the default standard of competence (Lee *et al*. 2013, 2023, Helmer *et al*. 2017, Smith 2021, Strauss *et al*. 2023).

The limited pool of peer reviewers with lived Indigenous experience and an understanding of Indigenous knowledges is a direct and indirect result of historic and ongoing racist colonial policies and remains a significant challenge in review selection (Lee *et al*. 2013, Helmer *et al*. 2017, Strauss *et al*. 2023). Over-reliance on this small group overburdens Indigenous, racialized and marginalized reviewers but can also enable journals to claim diversity or representation without addressing the systemic bias in their recruitment and selection practices (Lee *et al*. 2013, 2023, Helmer *et al*. 2017, Tennant *et al*. 2017, Strauss *et al*. 2023).

Further, many instances of what is labelled ‘peer review bias’ are more accurately understood as epistemic mis-qualification: situations in which reviewers are assigned to Indigenous-focused work despite lacking the conceptual, methodological, cultural, or ethical expertise required to assess it competently (Lee *et al*. 2013, 2023, Tennant *et al*. 2017, Strauss *et al*. 2023). This is particularly evident when Indigenous methodologies are evaluated against Euro-Western criteria of rigour, validity, or generalizability that are misaligned with Indigenous epistemologies and research purposes (Smith 2021, Watego 2021a, 2021b, Lee *et al*. 2023). In these cases, harm arises from prejudice but also from journals treating Euro-Western training and methodological traditions as universally sufficient to judge Indigenous methodologies and knowledges (Lee *et al*. 2013, 2023, Smith 2021).

In these contexts, Indigenous knowledges are frequently positioned as supplementary sources of contextual information rather than legitimate forms of evidence in their own right. Such positioning reinforces a hierarchy in which Euro-Western knowledge systems define the standards against which all other forms of knowledge are assessed, legitimized, and published (Lee *et al*. 2023).

Adding to this challenge, the ongoing Colonial Load (Box 1) or ‘minority tax’ experienced by potential Indigenous and racialized peer reviewers further limits availability and contributes to inequities in selection (Hirshfield and Joseph 2012, Rodríguez *et al*. 2015, Gaudry and Lorenz 2018) (see Box 1). Colonial Load refers to the cumulative, often invisible, labour, responsibilities, and emotional toll imposed on Indigenous peoples by colonial systems and institutions. This is work that is essential for those systems to function (Gaudry and Lorenz 2018). Yet it is typically unpaid, unrecognized, and disproportionately borne by Indigenous scholars and community members. Indigenous scholars face disproportionate, unpaid demands, including educating colleagues on racism, advising on culturally safe and appropriate methods, representing Indigenous perspectives in institutional forums, mentoring, and fulfilling ceremonial and community obligations (Rodríguez *et al*. 2015, Gaudry and Lorenz 2018). These demands, rarely recognized in workload allocations, intersect with reviewer selection and editorial decision-making, shaping what is deemed ‘credible’ research (Rodríguez *et al*. 2015, Gaudry and Lorenz 2018). By repeatedly privileging Euro-Western designs and relying on a narrow set of methodological norms, journals further constrain which Indigenous methodologies are seen as legitimate, fundable, and publishable (Rodríguez *et al*. 2015, Gaudry and Lorenz 2018, Smith 2021). By disproportionately selecting the same small group of Indigenous reviewers, journals can inadvertently limit the range of Indigenous methodologies deemed ‘credible’ or ‘valid’, shaping what knowledges are accepted into the academic record and reinforcing existing racialized hierarchies.

Box 1 Colonial Load The Colonial Load is not limited to these explicit tasks and is both expansive and routinely rendered invisible, including:

1. Addressing, mitigating, and resolving racism and colonial structures to actively promote anti-racism,
2. Building and maintaining connections between non-Indigenous and Indigenous peoples or communities, often through sharing personal social and kinship networks,
3. Hosting Indigenous communities, Elders, and Knowledge Keepers in institutional or community spaces,
4. Serving as the Indigenous representative on local, regional, national or international committees, governance bodies, or policy panels,
5. Providing Indigenous-specific representation on Human Research Ethics Committees (HREC), Research Ethics Boards (REB) and Indigenous Research Ethics Boards (IREB),
6. Mentoring Indigenous and non-Indigenous staff, students, and community members outside formal institutional programmes,
7. Conducting land acknowledgements, ceremonial openings, and culturally significant practices in professional or public settings,
8. Participating in Reconciliation committees, groups or initiatives, and
9. Fulfilling obligations and responsibilities defined by community protocols, cultural responsibilities, or community needs and priorities.

*Sources:* (Rodríguez *et al*. 2015, Gaudry and Lorenz 2018, Smith 2021, Walter and Suina 2023).

Indigenous scholars often carry these responsibilities alongside personal and community obligations not reflected in their formal roles or considered during publication and grant assessments, resulting in reduced capacity for research outputs (Gaudry and Lorenz 2018, Smith 2021, Lee *et al*. 2023, Maddox and Morton Ninomiya 2025). This reduction in available reviewers can compromise the objectivity, fairness, quality, and epistemic breadth of the peer review process and skew the evaluation of Indigenous research, proposals, and awards when assessed through predominantly non-Indigenous, Euro-Western frameworks (Gaudry and Lorenz 2018, Smith 2021, Lee *et al*. 2023, Strauss *et al*. 2023, Maddox and Morton Ninomiya 2025).

To address these challenges, some journals have begun including of Indigenous leadership, including senior editors and adopting specific criteria for selecting peer reviewers for Indigenous-focused articles (The Lowitja Journal 2026). Reviewers of Indigenous research must possess expertise in the article’s scientific or methodological focus, including the ability to assess its relevance and respect for the specific communities represented (The Lowitja Journal 2026). Reviewers should understand and respect Indigenous knowledge systems, methods, and context, including the cultural context and this should be a clear criterion in reviewer selection. Additionally, Indigenous editors are well-versed in the importance of Indigenous perspectives and knowledge frameworks. They can oversee the peer review of Indigenous research (Lee *et al*. 2013, 2023, Moreton-Robinson 2017, Lovett *et al*. 2019, Strauss *et al*. 2023, The Lowitja Journal 2026). This helps ensure that reviews are conducted through a culturally attuned and respectful lens. These structural interventions can help mitigate bias and better support the inclusion of Indigenous knowledge within academic discourse (Moreton-Robinson 2017, Strauss *et al*. 2023, The Lowitja Journal 2026).

#### Disrupting racism

Journals can develop reviewer pools inclusive of Indigenous scholars and reviewers trained in Indigenous epistemologies and cultural safety (Lovett *et al*. 2019, Lee *et al*. 2023, The Lowitja Journal 2026). Incorporating cultural safety training, including anti-Indigenous racism and anti-racism training, for all reviewers. This should include establishing guidelines to match Indigenous-focused research (see Box 2) with reviewers familiar with Indigenous knowledge systems can help create a more balanced, informed, and culturally safe review process as well as community-developed evaluative frameworks (e.g. Indigenous-led integrity tests such as the *Well Living House appraisal tool* or *The Blackfulla Test: 11 reasons that Indigenous health research grant/publication should be rejected*).

Box 2 Embedding Indigenous accountability at submission—an example from the *Canadian Journal of Public Health* The *Canadian Journal of Public Health* (*CJPH*) requires that all authors answer the following questions as part of the manuscript submission process:

- Are First Nations, Inuit, Métis, and/or Indigenous Peoples or populations a focus of this submission? Yes/No
- If yes, were the relevant Indigenous Peoples or populations engaged in the study and/or preparation of this submission? Yes/No
- If yes, please summarize how the relevant Indigenous Peoples or populations have been engaged as individuals and collectively in the study and/or preparation of this submission (2000 character limit).

*Sources:* (Hayward *et al*. 2021, The Canadian Journal of Public Health 2026)

### Review criteria and instructions

Reviewers are frequently given standard evaluation criteria focused on aspects like academic rigour, originality, and significance, which are typically defined within a Euro-Western and Eurocentric framework. This approach often fails to recognize the validity of Indigenous methodologies, sidelining them as ‘non-scientific’ or ‘unorthodox’ (Lovett *et al*. 2019, Lee *et al*. 2023, Strauss *et al*. 2023).

#### Disrupting racism

Journals can introduce evaluation criteria that explicitly acknowledge and value non-Euro-Western frameworks, such as decolonizing frameworks and community engagement, which can help to better balance power dynamics (Smith 2021, Lee *et al*. 2023). Providing guidelines or checklists for evaluating Indigenous methodologies, and training reviewers to assess Indigenous research on its own terms, can broaden what is considered ‘rigorous’ science (Lovett *et al*. 2019, Lee *et al*. 2023, Strauss *et al*. 2023).

### Initial manuscript screening

Manuscripts are often screened before peer review to assess their suitability, a process through which Indigenous-focused research is frequently dismissed as ‘niche’ or as lacking ‘relevance’ to so-called ‘whole populations’ or mainstream audiences (Smith 2021, Lee *et al*. 2023, Strauss *et al*. 2023). In these early screening decisions, rigour is often implicitly assessed through alignment with Euro-Western epistemologies and methodological norms, prior to external review. Such judgements are not neutral. They reproduce a cycle of exclusion in which Indigenous research and evaluation are systematically devalued, marginalized, and actively sidelined within academic publishing (Smith 2021, Lee *et al*. 2023, Strauss *et al*. 2023).

#### Disrupting racism

Establish, implement, monitor, and evaluate policies that explicitly include Indigenous knowledges and prioritize diversity in subject matter (Smith 2021, Lee *et al*. 2023). Journals can implement a ‘relevance’ checklist that includes the significance of Indigenous research and evaluation in informing and advancing fields and enhancing overall scientific understanding and rigour (Watego 2021a, 2021b, The Lowitja Journal 2026). A recurring manifestation of epistemic mis-qualification occurs when reviewers invoke dominant evidence hierarchies, including the elevation of meta-analyses, systematic reviews, and RCTs as the default or highest standard of rigour, without recognizing that these hierarchies are themselves products of particular epistemological traditions and value systems. Such demands reflect methodological imperialism, privileging Euro-Western hierarchies of evidence over Indigenous-led, community-governed, and relational research designs, regardless of their ethical appropriateness or demonstrated effectiveness (Lovett *et al*. 2019, Smith 2021, Lee *et al*. 2023, Strauss *et al*. 2023). A further problematic criterion is study generalizability, which can be in tension with resisting Eurocentric essentialization of the rich diversity of Indigenous nationhood and holding up the integrity of specific Indigenous knowledges and practices which are commonly rooted in local contexts (Smith 2021, Lee *et al*. 2023).

### Reviewer feedback

Reviewer feedback often reflects inherent biases, with Indigenous methodologies dismissed as ‘anecdotal’ or ‘not objective’. The reviewers may unconsciously judge the work against a White standard or ignore the cultural nuances essential to Indigenous research (Smith 2021, Lee *et al*. 2023). This bias can lead to recommendations for extensive revisions or outright rejection (Smith 2021, Lee *et al*. 2023, Strauss *et al*. 2023).

#### Disrupting racism

Journals can require reviewers to declare potential biases or limitations in their understanding of Indigenous methodologies before completing their review (Lee *et al*. 2013, 2023, Smith 2021, Strauss *et al*. 2023). Incorporating reflexivity statements and feedback forms that ask reviewers to self-reflect on potential biases can help prompt reviewers to be more mindful of their positionality, acknowledging, mitigating, and reducing bias in feedback (Lee *et al*. 2013, 2023, Smith 2021, Strauss *et al*. 2023).

### Editorial decision-making

Final editorial decisions often reflect the dominant scientific paradigm, which may privilege Euro-Western methodologies and knowledges, leading to the rejection of Indigenous approaches perceived as insufficiently ‘evidence-based’ (Lee *et al*. 2013, 2023, Smith 2021, Strauss *et al*. 2023). Editorial interpretation of peer review reports is itself shaped by epistemic positioning. Where editors lack the cultural, historical, or epistemic literacy to recognize racism or deficit framing, responsibility is often shifted onto Indigenous authors to identify, explain, and justify why particular reviewer comments are harmful. This displacement of labour reflects epistemic mis-qualification at the editorial level and reproduces unequal and racialized burdens within the peer review process (Lee *et al*. 2013, 2023, Smith 2021, Strauss *et al*. 2023).

Editorial decision-making is not a neutral aggregation of reviewer opinions. Editors actively interpret, prioritize, and legitimize reviewer feedback, and therefore play a decisive role in either interrupting or reproducing racism in peer review (Lee *et al*. 2013, Smith 2021, Strauss *et al*. 2023). When editors fail to critically assess reviews for racialized language, deficit framing, or epistemic mis-qualification, they effectively endorse these harms through institutional authority. Editors and reviewers without adequate training in Indigenous research, methodologies, or ethics may defer to reviewer commentary framed as ‘technical’ or ‘objective’, reinforcing systemic exclusion (Lee *et al*. 2013, Smith 2021, Strauss *et al*. 2023).

#### Disrupting racism

Journals can establish an Indigenous board or consult Indigenous experts for decisions involving Indigenous research (Lovett *et al*. 2019, Smith 2021, Strauss *et al*. 2023, Maddox and Morton Ninomiya 2025, The Lowitja Journal 2026).

Implementing a policy to review editorial decisions for potential biases, particularly in the case of Indigenous-focused manuscripts, can enhance accountability and inclusivity (Lovett *et al*. 2019, Smith 2021, The Lowitja Journal 2026).

### Post-publication assessment and repercussions

Published research and evaluations often shape funding and policy decisions, creating long-term impacts on public health narratives (Legg *et al*. 2021, Smith 2021, Lee *et al*. 2023). When Indigenous knowledges are oppressed, marginalized, devalued, or excluded in academic journals, it limits understanding and leads to policies that overlook Indigenous needs and priorities, perpetuating a cycle of oppression and marginalization (Smith 2021, Watego 2021a, 2021b, Lee *et al*. 2023).

#### Disrupting racism

Journals can highlight Indigenous-led research in their publication strategies and include Indigenous methodologies and frameworks as legitimate contributions to scientific discourse (Watego 2021a, 2021b, Lee *et al*. 2023, The Lowitja Journal 2026). Establishing metrics that value community impact and engagement can shift the focus from Euro-Western-centric academic standards to broader, inclusive measures of success and academic excellence (Lovett *et al*. 2019, Smith 2021, Lee *et al*. 2023, Maddox and Morton Ninomiya 2025).

Addressing these issues within peer review is one necessary intervention in dismantling anti-Indigenous biases in academic publishing, but it is not sufficient on its own (Lee *et al*. 2013, Piper *et al*. 2022, Strauss *et al*. 2023). Current systems in academic health sciences journals often operate within capitalist frameworks that prioritize profitability over accountability to the communities their research affects. Embedding culturally safe, relational, and structurally accountable peer review practices is essential to mitigating the dominance of settler colonial language and discourse and to respecting Indigenous KT approaches (Lee *et al*. 2013, Piper *et al*. 2022, Strauss *et al*. 2023). However, through coordinated structural reform across publishing systems academic publishing can begin to interrupt the systemic racism embedded within its foundations (Lee *et al*. 2013, Piper *et al*. 2022, Strauss *et al*. 2023).

This contributes to a narrow view of knowledges, research, and evaluation practices, limiting the inclusivity of the academic discourse and perpetuating racism and systemic discrimination in the peer review process. This actively hinders research and academic excellence (Smith 2021, Piper *et al*. 2022, Lee *et al*. 2023).

## Epistemic mis-qualification vs. peer review bias

Understanding and (re)framing peer review bias as epistemic mis-qualification shifts responsibility away from a sole focus on individual intent and towards editorial systems that routinely normalize the assignment of unqualified reviewers to Indigenous research (Ha Shilth 2013, Lee *et al*. 2013, Strauss *et al*. 2023). This (re)framing is not intended to absolve individual reviewers or editors of responsibility but to clarify how individual actions are enabled, legitimized, and reproduced by institutional processes and decision-making structures. This framing makes clear that harm can occur in the absence of overt hostility, and that reviewer training, selection criteria, and accountability mechanisms are central to effective anti-racist reform (Lee *et al*. 2013, Strauss *et al*. 2023).

Although this Perspective focuses on Indigenous scholarship, epistemic mis-qualification may also help explain how academic publishing marginalizes other forms of knowledge(s) and expertise that sit outside dominant disciplinary, methodological, or cultural norms.

Understanding these dynamics has relevance beyond Indigenous research and may help journals identify and address broader patterns of exclusion within peer review systems (Lee *et al*. 2023).

That said, implicit and explicit bias in the assessment of manuscripts under peer review remain significant barriers to advancing research and academic excellence (Lee *et al*. 2013, 2023, Strauss *et al*. 2023). Recognizing epistemic mis-qualification does not diminish the role of individual bias, rather, it situates such bias within systems that often fail to identify, challenge, or correct it. Implicit biases, which are unconscious and pervasive, are particularly insidious because they often go unrecognized by reviewers yet shape judgements, recommendations, and editorial outcomes, reinforcing established inequities (Lee *et al*. 2013, 2023, Strauss *et al*. 2023). Evidence shows that implicit bias is common across diverse fields, including health, where implicit bias is well documented across health systems and can shape clinical judgement and care, including for Indigenous peoples (Paradies 2006, Lee *et al*. 2013, Strauss *et al*. 2023). Within peer review, these same dynamics can influence how evidence is interpreted, weighted, and ultimately legitimized (Lee *et al*. 2013, Strauss *et al*. 2023). To help address this, implicit bias training is essential for peer reviewers and editors alike, to raise awareness of how biases can infiltrate assessments and contribute to discriminatory practices within peer review. Further, anti-racism and cultural safety education, including structured reflection on implicit bias, is necessary but insufficient without enforceable structural reform (Paradies 2006, Lee *et al*. 2013, Strauss *et al*. 2023).

Reviewers, consciously or unconsciously, hold assumptions, biases, and stereotypes that influence peer review assessment, often grounded in a Whitewashed evidence base and reproduced through everyday academic norms (Paradies 2006, Lee *et al*. 2013). These assumptions do not operate in a vacuum. They are actively reinforced when editorial systems accept, act upon, or fail to challenge racially biased reviews. For example, in response to a journal’s call for research on racism in health systems, peer reviewers described terms such as ‘colonial histories’, ‘Indigenous peoples’, and ‘health inequities’ as ‘catchphrases’ that should not be used in academic literature (Paradies 2006, Lee *et al*. 2013, Strauss *et al*. 2023). These terms were dismissed as inappropriate, disregarding the deep historical, political, and epistemic realities they describe.

However, racism within the peer review process does not end with reviewer commentary or editorial interpretation (Paradies 2006, Lee *et al*. 2013, Strauss *et al*. 2023). It can also be reproduced within author teams as they decide how, or whether, to respond to harmful feedback. Importantly, the (re)production of racism within the publication process is not confined to reviewers or editors. Author assumptions, biases, and stereotypes can also perpetuate racism during the author response stage, particularly within mixed Indigenous and non-Indigenous author teams responding to racist peer review (Paradies 2006, Lee *et al*. 2013, 2023, Strauss *et al*. 2023).

This shift from reviewer bias to (co) author dynamics highlights how racism can be sustained even after it has been identified, depending on how power operates within author teams (Paradies 2006, Lee *et al*. 2013, Strauss *et al*. 2023).

The harms of anti-Indigenous racism in peer review extend beyond reviewer comments, editorial decisions, and publication outcomes. Anticipation of racism can also shape what Indigenous scholars choose to write, how they frame their arguments, and whether particular critiques are expressed at all. Dotson (2011) describes this phenomenon as ‘testimonial smothering’, whereby individuals limit, modify, or withhold testimony because they reasonably anticipate misunderstanding, dismissal, or prejudice from their audience. Extending Dotson’s (2011) concept of testimonial smothering to academic publishing, we suggest that repeated experiences of racism, epistemic mis-qualification, and hostile peer review may lead Indigenous scholars to avoid particular topics, moderate language relating to colonialism, racism, or sovereignty, remove Indigenous theoretical framing, or alter interpretations to align with anticipated reviewer expectations.

These adaptations may improve the likelihood of publication but can simultaneously constrain Indigenous intellectual freedom and shape what knowledge ultimately enters the scholarly record. When Indigenous scholars anticipate that Indigenous methodologies, analyses, or community-held knowledges will be judged against dominant evidence hierarchies, testimonial smothering may occur. Authors may moderate, (re)frame, or omit Indigenous theoretical perspectives in anticipation of reviewer expectations, thereby linking epistemic exclusion to the self-censorship and marginalization of Indigenous knowledges.

Testimonial smothering may also influence where Indigenous scholars choose to publish. Following experiences of racism, epistemic mis-qualification, or culturally unsafe peer review, some Indigenous researchers may avoid submitting to particular journals, editors, or publishing environments altogether. While often invisible within publication metrics, such decisions represent an important form of institutional disengagement that can further limit the presence of Indigenous scholarship within mainstream academic literature. Consequently, anti-Indigenous racism shapes what is published, but also whose knowledge enters academic debate, which questions are pursued, and which perspectives remain absent from the scholarly record.

### Author response dynamics

Co-authors, particularly non-Indigenous or non-racialized authors, have a responsibility to support colleagues experiencing racism in peer review, rather than retreating into silence, risk aversion, or institutional self-protection (Gaudry and Lorenz 2018, Lee *et al*. 2023). In some cases, non-Indigenous (co)authors have amplified harm by reframing the raising of racism as an ‘ethical’ breach, thereby deflecting attention away from racism itself and further entrenching power imbalances (Gaudry and Lorenz 2018, Smith 2021, Lee *et al*. 2023, Strauss *et al*. 2023).

Furthermore, (co)authors can be complicit in perpetuating these biases, actively maintaining existing power structures and the status quo (Moreton-Robinson 2017). To illustrate how these dynamics operate in practice, we draw on anonymized, real-world experiences shared by Indigenous researchers (Gaudry and Lorenz 2018). These examples are included as analytic illustrations of recurring patterns within academic publishing, not as personal exposé or attribution of intent to identifiable individuals. Where positionality is referenced (e.g. seniority or career stage), this reflects observed power dynamics within author teams rather than confirmed racial, cultural, or Indigenous identity. In some instances, the racial or Indigenous identity of the individuals involved was unknown or not central to the analytic point (Strauss *et al*. 2023, Maddox and Morton Ninomiya 2025).

For instance, in one case, a senior author, when faced with racist rhetoric in peer review, responded, ‘I PERSONALLY HAVE DECIDED TO LET IT GO…’ and ‘…NOT TO ROCK THE BOAT TOO MUCH’, effectively signalling acceptance of racialized harm within academic spaces. Another example involved an early-career author relinquishing leadership and suggesting they would step off a paper when a racism complaint was raised. These responses are not presented as personal failings, but as illustrative of how institutional pressure, risk aversion, and unexamined bias can reproduce harms (Lee *et al*. 2013, Gaudry and Lorenz 2018, Strauss *et al*. 2023, Maddox and Morton Ninomiya 2025). Such examples demonstrate how racism can be sustained through silence, minimization, or withdrawal, regardless of the stated intentions of those involved (Lee *et al*. 2013, Gaudry and Lorenz 2018, Strauss *et al*. 2023, Maddox and Morton Ninomiya 2025).

These are not neutral acts. They function as forms of complicity within racialized academic systems (Lee *et al*. 2013, Gaudry and Lorenz 2018, Strauss *et al*. 2023). Such responses signal to journals, authors, researchers, and wider academic communities that racism is tolerable if it preserves collegiality, institutional relationships, or personal comfort. This reinforces existing power hierarchies and sustains a status quo that privileges Whiteness (Lee *et al*. 2013, 2023, Gaudry and Lorenz 2018, Strauss *et al*. 2023).

Implicit bias, even when not overt, can subtly skew reviews. Returning to reviewer commentary specifically, consider a peer reviewer comment such as: ‘I think it would also be appropriate to note that 40% smoking prevalence is an appallingly high figure…’ This example is drawn from reviewer feedback and is included to illustrate how everyday evaluative language within peer review can reproduce harm (Lee *et al*. 2013, 2023, Katz *et al*. 2020). While the intention behind such feedback may appear benign or descriptive, it perpetuates a deficit discourse around Indigenous health, framing Indigenous populations as inherently flawed or deviant. This deficit framing has long characterized relationships between Indigenous and non-Indigenous peoples, ignoring contextual, structural, commercial, and historical determinants and reinforcing colonial narratives. Deficit framing is not simply an unfortunate choice of words; it reflects and reproduces racialized logics that justified colonial governance, stratification, and violence (Lee *et al*. 2013, 2023, Katz *et al*. 2020). These discourses are embedded within dominant evidence hierarchies and causal frameworks, including conventional epidemiological standards that prioritize individualized risk and moralized interpretation over structural analysis. Such language contributes to disparities in how research is assessed, interpreted, and valued (Katz *et al*. 2020, Lee *et al*. 2023).

The Bradford Hill causality criteria, a standard in scientific assessment, exemplify how supposedly objective frameworks can exclude Indigenous peoples and knowledges (Schünemann *et al*. 2011). However, when interpreted narrowly, these frameworks may privilege linear exposure–outcome relationships, experimental designs, and individual-level risk factors. This can make it more difficult for research that foregrounds relational, historical, and structural determinants of health, including colonization, structural violence, and commercial determinants, to be recognized as providing robust causal insight. The issue is therefore not the causal frameworks themselves, but how they are interpreted and applied within dominant evidence hierarchies. In practice, these epistemic assumptions can surface both through formal methodological critique and through everyday reviewer language that moralizes and decontextualizes Indigenous health data (Schünemann *et al*. 2011, Lovett *et al*. 2019, Katz *et al*. 2020, Lee *et al*. 2023, Maddox and Morton Ninomiya 2025).

More broadly, these dynamics reflect the influence of EBP frameworks and associated hierarchies of evidence that have become dominant within health sciences. While EBP has contributed substantially to improving transparency, consistency, and methodological rigour within health sciences, scholars have argued that the dominant hierarchies of evidence underpinning EBP are neither neutral nor universal. Rather, they reflect particular epistemological assumptions about what constitutes evidence, causality, validity, and rigour (Lee *et al*. 2023).

Luke and colleagues (2022) argue that the application of evidence hierarchies developed within Euro-Western traditions can create ethical tensions in Indigenous health and social research because they privilege particular methodological traditions and definitions of rigour. In doing so, Indigenous ways of knowing, community-held knowledge, relational accountability, oral traditions, and lived experience may be treated as lower forms of evidence or supplementary sources of knowledge rather than rigorous forms of evidence in their own right.

Importantly, this is not an argument against rigour, evidence, critical appraisal, or methodological standards. Rather, it raises questions about how rigour is defined, who has been empowered to define it, and whether existing standards adequately recognize diverse epistemological traditions (Luke *et al*. 2022, Lee *et al*. 2023).

When reviewers and editors uncritically apply dominant evidence hierarchies, Indigenous scholarship may be disadvantaged not because it lacks rigour, but because prevailing definitions of rigour are often rooted in Euro-Western assumptions that do not fully recognize Indigenous epistemologies, methodologies, and forms of evidence (Luke *et al*. 2022, Lee *et al*. 2023).

For example, peer reviewers have described Indigenous smoking prevalence as ‘appallingly high’, framing population-level data as evidence of failure or deviance rather than as the predictable outcome of structural violence, colonization, and commercial targeting (Katz *et al*. 2020, Smith 2021). While such comments may appear descriptive or value-neutral, they reproduce deficit-based logics that align with Euro-Western causal frameworks prioritizing individualized risk, linear explanation, and moral judgement over historical, political, and commercial determinants (Paradies 2006, Katz *et al*. 2020, Smith 2021).

Similarly, Indigenous-led research demonstrating the effectiveness of community-governed smoking cessation initiatives, grounded in cultural revitalization, relational accountability, and place-based practice, may be dismissed as lacking ‘causal certainty’ when assessed primarily through conventional linear exposure–outcome models or experimental hierarchies of evidence (Schünemann *et al*. 2011, Lovett *et al*. 2019, Smith 2021, Piper *et al*. 2022, Maddox and Morton Ninomiya 2025).

In Indigenous-led research contexts, causality is often understood through sustained community observation, intergenerational knowledge, consistency across settings, and culturally grounded mechanisms of change, forms of evidence that are highly meaningful and actionable for Indigenous communities, yet frequently discounted or dismissed as insufficient or ‘anecdotal’ within Euro-Western causal hierarchies (Lee *et al*. 2023). Claims of rigid objectivity, stripped of cultural and historical context, have become entrenched in academic systems, reinforcing biases that marginalize Indigenous voices and privilege Euro-Western evidence hierarchies (Watego 2021a, 2021b, Lee *et al*. 2023, Strauss *et al*. 2023).

The harms described across reviewer bias, epistemic mis-qualification, editorial complicity, and co-author dynamics do not end at publication. They shape the evidence base that informs clinical guidelines, policy decisions, funding priorities, and service delivery (Lee *et al*. 2013, 2023, Legg *et al*. 2021, Smith 2021, Watego 2021a, 2021b, Strauss *et al*. 2023, Maddox and Morton Ninomiya 2025). Evidence highlights detrimental effects on Indigenous health and wellbeing outcomes extending far beyond the publication process itself, including where clinical non-adherence to guidelines disproportionately affects Indigenous patients and reinforces health inequities (Lee *et al*. 2013, 2023, Watego 2021a, 2021b, Strauss *et al*. 2023, Maddox and Morton Ninomiya 2025). Biases that marginalize Indigenous knowledges at the point of peer review influence what is recognized as valid evidence, with downstream consequences for care quality, access, and outcomes (Smith 2021, Watego 2021a, 2021b, Lee *et al*. 2023).

Addressing epistemic mis-qualification and implicit bias in peer review is a scholarly concern but also a public health imperative. When racialized evidence hierarchies are normalized within journals, they are reproduced across the health system, shaping clinical practice, performance metrics, research funding priorities, and policy decisions. Failure to intervene at this upstream stage risks perpetuating inequities across the entire health system. Reform is therefore required to protect authors but also to protect the communities whose lives are shaped by the knowledge that peer review legitimizes (Lee *et al*. 2023, Strauss *et al*. 2023). Journals must adopt culturally safe, structurally accountable peer review systems that include anti-racism training, editorial accountability, and recognition of Indigenous epistemologies and methodologies. By doing so, academic publishing can begin to dismantle Euro-Western epistemic dominance and reduce the broader harms that flow from a Whitewashed evidence base, fostering environments in which Indigenous knowledge systems are respected, protected, and valued. Confronting racism in peer review is therefore essential not only for academic integrity but also for achieving equitable, ethical, and effective health promotion (Lee *et al*. 2023, Strauss *et al*. 2023, Maddox and Funnell 2024, Sirleaf 2025).

## Challenges to addressing racism in peer review: improving academic excellence and trust in science?

This Perspective recognizes racism in peer review and its pervasive nature, and seeks to advance science, research and academic excellence by improving the peer review processes and actively holding the academy to account. However, the challenge is complex as racism and coloniality manifest in diverse ways (Lee *et al*. 2023, Strauss *et al*. 2023, Sirleaf 2025).

Racism and colonization in academic publishing are rarely singular acts. They are upheld by a complex system of editorial governance, peer review practices, and knowledge valuation that may or may not be influenced by powerful industry actors (Lee *et al*. 2013, 2023, Smith 2021, Strauss *et al*. 2023, Sirleaf 2025).

Academic journals and peer review systems are not insulated from these broader political and commercial forces, particularly in fields such as tobacco and nicotine research, where industry interests have a long history of shaping scientific norms, debates, and boundaries of legitimacy (Lee *et al*. 2013, 2023, Smith 2021, Strauss *et al*. 2023, Sirleaf 2025).

For example, the Health Equity Network Advisory Committee for the Society for Research on Nicotine and Tobacco (SRNT) recently highlighted concerns (Rose *et al*. 2024) about how the commercial Tobacco and Nicotine Industry and its affiliates undermine public health through influence over research agendas, evidence production, and scientific discourse (Rose *et al*. 2024). This influence operates at the level of conference programming and funding streams, but it also shapes the assumptions reviewers and editors bring to manuscript assessment (Rose *et al*. 2024). When industry-aligned framings are normalized within a field, they can subtly inform what is considered methodologically rigorous, policy-relevant, or appropriately framed within journal submissions (World Health Organization 2003, Legg *et al*. 2021, Piper *et al*. 2022, Lee *et al*. 2023, Strauss *et al*. 2023, Maddox and Morton Ninomiya 2025). In peer review contexts, this may manifest as resistance to structural or rights-based critiques of industry power, scepticism towards decolonial or Indigenous sovereignty frameworks, or the privileging of research questions that align with market-based or harm-reduction logics (United Nations 2007). These dynamics are directly relevant to academic journal peer review, as they shape which research questions are seen as legitimate, which methodologies are privileged, and how critiques of industry power, particularly those grounded in Indigenous rights, sovereignty, and lived experience, are assessed, legitimized, and published by reviewers and editors (Lee *et al*. 2023, Strauss *et al*. 2023, Maddox and Morton Ninomiya 2025). In this way, peer review becomes a site where broader political and commercial struggles over legitimacy are enacted through seemingly technical decisions about rigour, relevance, and framing (Lee *et al*. 2023, Strauss *et al*. 2023).

Specifically, they emphasized that the commercial interests of the Tobacco and Nicotine Industry and their affiliates are fundamentally at odds with the human right to health, including the rights of Indigenous peoples and children to enjoy the highest attainable standard of health (World Health Organization 2003, United Nations 2007, Rose *et al*. 2024). When peer review processes fail to recognize or critically interrogate these conflicts, they risk reproducing industry-aligned framings while marginalizing Indigenous-led, rights-based, and decolonial scholarship within the academic record (United Nations 2007, Legg *et al*. 2021, Piper *et al*. 2022, Lee *et al*. 2023).

In contrast, a published industry perspective stated that it was ‘…simply raising questions about the implications of excluding anyone with an affiliation with the commercial tobacco and nicotine industry from attending and presenting their science at the annual SRNT conference and/or publishing within NTR’. The response uses ostensibly neutral language, such as appeals to ‘openness’, ‘transparency’, or ‘fairness’, that can mask and legitimize the continued inclusion of actors whose interests conflict with public health (World Health Organization 2003, Katz *et al*. 2020, Legg *et al*. 2021, Lee *et al*. 2023, Weiss 2024). This framing, articulated through references to ‘open, transparent processes with guard rails’, signals balance and inclusivity while shifting attention away from profound power asymmetries and the ongoing historical and contemporary harms associated with the commercial Tobacco and Nicotine Industry (Katz *et al*. 2020, Legg *et al*. 2021, Lee *et al*. 2023, Weiss 2024).

Such forms of neutral-sounding language can function to normalize industry presence within academic spaces by recasting exclusion as intolerance and regulation as censorship, rather than as necessary protection of public health, human rights, and Indigenous rights (United Nations 2007, Smith 2022). This framing may contribute to creating unsafe spaces at conferences and within journals and academia. At the same time, the industry continues to actively manufacture and market addiction, disease, and death around the world, disproportionately harming Indigenous and racialized peoples (World Health Organization 2003, Lee *et al*. 2023). Such examples can illustrate how structural racism in peer review intersects with broader systems of commercial and colonial power, reinforcing inequities under the guise of neutrality (Watego 2021a, 2021b, Lee *et al*. 2023).

Similarly, academic actors who claim to support anti-racism yet withdraw when faced with institutional pushback serve to extend and protect this system, whether consciously or not (Watego 2021a, 2021b). This example underscores that racism and colonization in academic publishing are rarely singular acts. Rather, they manifest through interlocking industry influence, institutional complicity, and the normalization of unsafe spaces, making them complex to dismantle (Smith 2021, Watego 2021a, 2021b, Lee *et al*. 2023).

While peer review cannot always address or mitigate racism or ethical concerns, it remains central to scientific publishing and research funding systems, with no widely accepted alternative currently available (Paradies 2006, Watego 2021a, 2021b, Lee *et al*. 2023, Strauss *et al*. 2023). However, recognizing racism and ongoing racialized structures and epistemic foundations that underpin peer review is critical to addressing and moving to anti-racist peer review processes that explicitly confront anti-Indigenous racism (Paradies 2006, Watego 2021a, 2021b, Lee *et al*. 2023, Maddox and Morton Ninomiya 2025).

It is also essential to recognize the role of (co)authors when incidents of racism occur. When authors remain silent, discourage formal complaints, or (re)frame incidents of racism as a misunderstanding or an ‘oversight’, they are not neutral actors, they are complicit (Watego 2021a, 2021b, Maddox and Morton Ninomiya 2025). Remaining silent or discouraging the naming of racism is not passive bystanders behaviour, it actively sustains a peer review system in which inequities are reproduced by inaction (Watego 2021a, 2021b, Maddox and Morton Ninomiya 2025). Such complicity functions as tacit endorsement of the status quo, signalling to editors, institutions, academic and broader societal networks that racism can proceed without consequence. More concerning are instances where (co)authors actively raise ‘ethical’ or ‘integrity’ concerns about the act of naming racism and fostering unsafe spaces, particularly for Indigenous and racialized peoples and communities. These moves deflect attention from the harm of racism itself, recentring Whiteness, and shifting the burden of justification on Indigenous and racialized authors and can weaponize institutional processes to protect dominant interests (Watego 2021a, 2021b, Lee *et al*. 2023, Strauss *et al*. 2023). This dynamic entrenches existing power imbalances and reinforces a peer review culture that privileges White comfort over Indigenous and racialized safety, truth-telling, and anti-racism best practice (Watego 2021a, 2021b, Piper *et al*. 2022, Strauss *et al*. 2023). The irony of raising ‘ethical’ or ‘integrity’ questions is not lost in such circumstances, nor is the work and colonial load generated by those raising such concerns and the burden of responsibility placed on Indigenous and racialized peoples.

Addressing racism in peer review is essential for the advancement of knowledges and research excellence, but also for upholding ethical obligations to Indigenous authors and communities (Paradies 2006, Smith 2021). Equity in peer review requires vigilance at every stage, from reviewer selection to editorial decisions to co-author dynamics and publication dissemination, ensuring that the process itself does not reproduce the very inequities some claim to challenge (Paradies 2006, Lee *et al*. 2013, 2023, Helmer *et al*. 2017, Smith 2021).

## Calls for action/wise practices and potential solutions

To address anti-Indigenous racism and other forms of discrimination in peer review, coordinated and enforceable actions are required at every level of the research and publishing ecosystem (Paradies 2006, Lee *et al*. 2023, Strauss *et al*. 2023, Maddox and Morton Ninomiya 2025). This requires both immediate interventions to prevent ongoing harms and sustained structural change to dismantle the systems that reproduce harms. This includes explicit commitments to resourcing and reciprocity within profit-generating publishing systems, including remuneration for Indigenous review labour and ensuring that Indigenous-led research is openly accessible to the Indigenous communities it concerns (Paradies 2006, Legg *et al*. 2021, Smith 2021, Strauss *et al*. 2023).

These recommendations are intentionally framed as structural and enforceable, rather than discretionary (see Table 3). In our experience and in the literature, existing institutional complaints, ethics, and misconduct pathways are ill-equipped to recognize, evidence, or respond to racism in peer review, particularly where harm is epistemic, cumulative, or relational rather than overt (Paradies 2006, Watego 2021a, 2021b, Strauss *et al*. 2023). In such contexts, responsibility is frequently shifted back onto the harmed individual, who is expected to meet an evidentiary threshold that institutions themselves have not defined, resourced, or culturally attuned their processes to assess. This produces a profound accountability gap across journals, institutions, and professional bodies, enabling harm to persist without consequence (Paradies 2006, Watego 2021a, 2021b, Piper *et al*. 2022, Strauss *et al*. 2023).

**Table 3:** Structural reforms to address racism and epistemic exclusion in peer review.

**1. Critical self-reflection and accountability**
All reviewers, editors, and (co)authors must engage in structured self-reflection on their positionality, privilege, and biases before participating in the peer review process, including critical reflection on their level of Indigenous-specific knowledge, expertise, and competence. This includes consideration of whether they understand, and can meaningfully operate within, the relevant Indigenous worldview, epistemologies, concepts, and research practices associated with the work under review, or whether additional expertise is required. Institutions and journals should mandate content-specific reflexivity statements for all reviewers and editorial decision-makers. When individuals are invited to review or make decisions on manuscripts that include Indigenous content, these statements must explicitly address the reviewer’s Indigenous knowledge, practice, and lived experience (or absence thereof), their epistemic positioning in relation to the research and communities involved, and any limitations in competence or perspective. These statements should be reviewed as part of editorial decision-making, rather than treated as symbolic disclosures, to ensure transparency about expertise, prevent epistemic mis-qualification, and support accountable and culturally safe review processes.
**2. Diversifying decision-making and reviewer pools**
Increase the proportion of Indigenous and other racialized peoples on editorial boards, Editor-in-Chief roles, grant review panels, and other decision-making positions, recognizing that this cannot be achieved without deliberate expansion, resourcing, and protection of the Indigenous workforce. This includes the creation of paid, properly resourced, and structurally supported positions, rather than reliance on unpaid, honorary, or goodwill-based labour. Such roles must be accompanied by clear workload expectations, remuneration, career progression pathways, and institutional accountability for retention, to avoid overburdening a small number of Indigenous scholars. Journals and institutions must invest in Indigenous workforce development, leadership pathways, mentoring, and career progression across academic publishing and research governance systems. In addition, non-academic Indigenous community members with relevant knowledge(s), practice, and lived experience should be recognized as legitimate contributors to peer review and editorial processes, with appropriate stipends or remuneration, including where community expertise is essential to assessing cultural, ethical, or contextual dimensions of the work, acknowledging their expertise as skilled labour rather than voluntary service. Establish standing Indigenous and community advisory boards to oversee reviews of Indigenous-focused work, recognizing that such boards must not rely on unpaid, informal, or goodwill-based labour. These boards should have formal decision-making authority, appropriate and ongoing resourcing, and clear accountability mechanisms, and be supported through paid roles, stipends, or honoraria that recognize Indigenous governance, community expertise, and lived experience as skilled labour. Membership should include Indigenous scholars and, where appropriate, non-academic Indigenous community members with relevant cultural, methodological, and contextual expertise, acknowledging that expertise in Indigenous research and knowledge systems is not confined to academic institutions. The establishment of such boards must be accompanied by sustained investment in Indigenous community governance, leadership, and participation, as well as dedicated human resources, administrative supports, and institutional infrastructure, to ensure durability, continuity, and authority, rather than symbolic inclusion.
**3. Training and capacity-building**
Implement mandatory anti-racism, cultural safety, and Indigenous methodologies training, including for Editors-in-Chief, associate editors, reviewers, funders, and institutional research committees, with regular refreshers and accountability mechanisms. Training must be complemented by routine, structured evaluations of peer review practice, including retrospective review of peer review reports and editorial decisions on Indigenous-focused manuscripts, to identify patterns of racialized language, deficit framing, epistemic mis-qualification, and inappropriate methodological demands. Where harmful practices are identified, journals should be required to document actions taken, which may include additional training, removal of reviewers or editors from Indigenous-focused work, or exclusion from reviewer pools where necessary. These evaluations should be ongoing rather than reactive, ensuring that accountability is embedded in standard editorial practice rather than dependent on individual complaints or disclosures of harm. Ensure training includes the history and ongoing impacts of colonialism in research, discipline-specific manifestations of epistemic racism, and the ethical obligations under UNDRIP. Develop and implement applied, context-based training that uses real-world, Indigenous-focused manuscript review case studies. Such training should require editors and reviewers to work through concrete examples of peer review reports, editorial decisions, and author responses, including identifying racist or deficit-based language, inappropriate methodological demands, epistemic mis-qualification, and culturally unsafe practices, and practising appropriate, accountable responses. Case-based training should be designed and led by Indigenous scholars and community experts, and grounded in Indigenous governance, knowledges, and lived experience, to ensure relevance, rigour, and cultural safety.
**4. Structural policy reforms**
Require journals to adopt and standardize explicit reviewer selection policies that prioritize methodological, epistemic, and relevant cultural expertise in addition to content and domain expertise. Establish transparent review allocation systems to avoid over-reliance on the same small pool of Indigenous reviewers, and to prevent the concentration of unpaid or uncompensated labour.
**5. Addressing complicity and (co)author responsibility**
Develop and implement clear guidelines on the responsibilities of non-Indigenous (co)authors when incidents of racism occur in the review process, including expectations for solidarity, public support, ethical advocacy, and resisting institutional retaliation. Integrate breach of these responsibilities into institutional and professional codes of conduct. Develop, implement, and enforce cross-institutional accountability frameworks to address racism and complicity in peer review, recognizing that codes of conduct are necessary but insufficient in the absence of enforceable mechanisms. These frameworks must include clear escalation pathways, independent adjudication processes, and protections against retaliation, and must not rely solely on individual complainants to substantiate harm in the absence of culturally competent investigative standards. Such frameworks should be capable of responding to harms that occur across journals, institutions, and professional roles, while remaining attentive to power asymmetries and prioritizing the protection of Indigenous and racialized scholars.
**6. Removing systemic barriers**
Identify and dismantle institutional practices that limit Indigenous access to reviewer or editorial roles, such as exclusionary eligibility criteria, unremunerated expectations, and invisible labour. Provide remuneration for Indigenous review work, acknowledging it as skilled labour rather than voluntary service, and ensuring parity with other forms of expert peer review. Remove economic barriers to participation and publication by requiring publishing houses and journals to implement automatic Article Processing Charge (APC) waivers or reduced fees for Indigenous-led research and for Indigenous scholars who undertake substantial peer review and editorial labour. These measures should be framed as structural equity and reciprocity within profit-generating publishing systems, rather than as discretionary concessions or individual compensation. Remove access barriers to knowledge dissemination by ensuring that Indigenous-led research is openly accessible to the Indigenous communities to whom the research relates, recognizing access to knowledge as a core component of Indigenous Data Sovereignty, ethical research practice, and reciprocity.
**7. Measuring and monitoring change**
Develop and publish annual diversity and equity audits of reviewer and editorial pools, review outcomes, and appeals processes, disaggregated where ethically appropriate and reported transparently. Where audits identify patterns of racialized harm, epistemic mis-qualification, or failure of editorial oversight, journals should be required to document corrective actions taken, which may include public acknowledgement through existing mechanisms such as published errata, editorial statements, or formal corrections to the scholarly record, where appropriate and consistent with procedural fairness and due process. Track the impact of published Indigenous research using metrics that value community benefit, knowledge sovereignty, and relational accountability, not just citation counts, and embed these measures into journal and institutional performance frameworks. Implement routine audits of peer review quality, not only reviewer diversity. These audits should examine patterns of racialized language, deficit framing, epistemic mis-qualification, and inappropriate methodological demands, and require journals to document how identified harms were addressed, escalated, or remediated, including whether concerns were referred to institutional or professional bodies where appropriate and whether repeat or egregious conduct triggered formal referral under established journal–institution reporting protocols.

Addressing anti-Indigenous racism in peer review requires more than procedural reform. It requires educational, cultural, and epistemic transformation across the publishing ecosystem. Academic journals are not simply gatekeepers of knowledge(s); they are powerful institutions that shape how generations of researchers, reviewers, and editors understand evidence, causality, rigour, expertise, and excellence.

The emergence of EBP provides an instructive example. Through coordinated editorials, educational series, reporting guidelines, and changes to publication standards, journals played a central role in (re)shaping how researchers understood and evaluated evidence. A comparable effort is now required to address anti-Indigenous racism, epistemic exclusion, and colonial assumptions embedded within academic publishing.

Journals, publishers, and professional societies should therefore invest in sustained educational initiatives, including editorials, commissioned commentaries, reviewer development resources, guidance documents, reporting standards, and continuing professional development opportunities focused on Indigenous methodologies, Indigenous Data Sovereignty, anti-racism, cultural safety, and epistemic justice. Such initiatives should not be viewed as optional diversity activities but as core components of research excellence, publication integrity, and scholarly accountability.

Without independent, enforceable accountability mechanisms, responsibility for identifying and evidencing racism continues to fall disproportionately on Indigenous scholars themselves, a burden that reproduces harm and undermines institutional claims of cultural safety (Paradies 2006, Watego 2021a, 2021b, Piper *et al*. 2022, Strauss *et al*. 2023).

While this Perspective focuses specifically on anti-Indigenous racism, many of the reforms proposed in this paper, including reviewer training, greater transparency, editorial accountability, diversification of reviewer pools, and recognition of multiple forms of evidence and expertise, are likely to improve fairness and rigour across peer review more broadly.

These reforms may also assist journals, institutions, and publishers to identify and address wider forms of bias, exclusion, and epistemic marginalization within academic publishing.

## Conclusion

Peer review is a health promotion issue. Peer review shapes the evidence, knowledge systems, and decision-making that influence policy, practice, and population health. Equity and anti-racism in peer review cannot be achieved through goodwill, individual intent, or symbolic inclusion alone (Paradies 2006, Piper *et al*. 2022, Strauss *et al*. 2023). It demands deliberate, enforceable structural change in reviewer recruitment, editorial decision-making, and (co)author accountability (Paradies 2006, Piper *et al*. 2022, Strauss *et al*. 2023). It is critical to recognize that no form of racism is benign.

Whether subtle or overt, implicit, or explicit, racism in peer review harms scholars, distorts knowledge production, diminishes the integrity of the scholarly record, perpetuates inequities, and reinforces the very structures this Perspective seeks to dismantle. Addressing racism therefore requires dismantling the norms, incentives, and processes that protect White comfort and institutional reputation. It also requires replacing them with systems that actively protect and support Indigenous and racialized scholars and knowledges, while centring safety, truth-telling, accountability, and sovereignty.

Peer review is likely to remain central to science and scholarly publishing because it is deeply embedded within academic governance and legitimacy, and because no widely accepted alternative exists (Piper *et al*. 2022, Lee *et al*. 2023, Strauss *et al*. 2023). This reality increases, rather than diminishes, our collective responsibility to ensure that peer review operates in ways that are just, transparent, accountable, and culturally safe, rather than exclusionary or harmful (Piper *et al*. 2022, Strauss *et al*. 2023). Anti-Indigenous racism in peer review does not merely determine which manuscripts are published. It shapes which questions are asked, which theories are developed, which findings are reported, which forms of evidence are recognized as legitimate, and ultimately what knowledge becomes possible within the academy (Lee *et al*. 2023). The lessons arising from this analysis also extend beyond Indigenous scholarship. Peer review systems that are more accountable, transparent, culturally safe, and epistemically diverse are likely to produce stronger science, more equitable publishing practices, and a richer evidence base for health promotion and public health action (Luke *et al*. 2022, Lee *et al*. 2023).

Transforming peer review requires more than new policies and procedures. Transforming peer review requires a sustained (re)examination of how academic communities are taught to understand evidence, rigour, expertise, and excellence. Confronting racism in peer review is not optional, nor peripheral to scholarly excellence. It is an ethical, political, and public health imperative that directly affects the wellbeing of scholars, the validity and credibility of the scientific record, and the capacity of research to serve Indigenous peoples and communities with integrity (Paradies 2006, Piper *et al*. 2022, Lee *et al*. 2023, Strauss *et al*. 2023). Transforming peer review is not only an academic reform but also a health promotion strategy that (re)shapes the knowledge systems determining whose lives are protected, prioritized, and valued (Legg *et al*. 2021, Lee *et al*. 2023, Strauss *et al*. 2023, Maddox and Morton Ninomiya 2025). Without intentional and sustained structural reform, peer review will continue to undermine science and justice, while (re)producing the very inequities that health promotion seeks to eliminate (Lee *et al*. 2013, 2023, Strauss *et al*. 2023, Maddox and Morton Ninomiya 2025). The future of equitable and excellent scholarship depends on diversifying who participates in peer review but also on transforming the structures that determine whose knowledge counts, whose voices are heard, and whose futures are imagined (Luke *et al*. 2022, Lee *et al*. 2023).

## Foundational to this perspective

This Perspective was led by Indigenous interests, needs, and rights as Indigenous peoples (R.M., S.F., M.K., and J.S.), consistent with the United Nations Declaration on the Rights of Indigenous Peoples (UNDRIP) and principles of ethical and wise practice (United Nations 2007, Smith 2021). It was conceptualized with Indigenous leadership and engagement, grounded in the everyday lived experience of racism in academic publishing, to support ethical practices that confront anti-Indigenous racism.

Relationality, our connections, responsibilities, and accountabilities to community, is central to this work (Moreton-Robinson 2017). It forms the epistemic scaffolding through which we generate knowledge in specific times, places, and lands (Moreton-Robinson 2017).

Privileging Indigenous logics of knowing allows us to remain anchored in who we are, who claims us, and how we are connected to Country. This is not simply a matter of identity, it is a matter of ontology, epistemology, and sovereignty (Moreton-Robinson 2017, Smith 2021, Maddox and Morton Ninomiya 2025).

Where non-Indigenous, non-racialized (co)authors are involved, their role in supporting Indigenous and racialized leadership must be active and accountable. This includes standing publicly with Indigenous and racialized colleagues when racism occurs, resisting pressures to minimize or dismiss harms, and rejecting self-protective withdrawal. Silence in these moments perpetuates harm and erodes trust in research relationships (Paradies 2006, Smith 2021, Piper *et al*. 2022).

As Indigenous scholars (R.M., S.F., M.K., J.S.), we bring to this work our academic experience but also our responsibilities as community members, mothers, children, uncles, aunties, bubus, grandparents, and kin. Immersed in these everyday realities, we critically reflect on racism in peer review, expose its racialized logics, and propose solutions grounded in sovereignty, solidarity, and justice.

## Data Availability

Not applicable.
